# WeChat-platform-based education and care program as a candidate approach to relieve anxiety, depression, and post-traumatic stress disorder in parents of pediatric and adolescent patients with osteosarcoma

**DOI:** 10.3389/fpsyg.2022.913940

**Published:** 2022-08-25

**Authors:** Jing Wu, Jie Meng, Honghe Li

**Affiliations:** Department of Orthopedic Surgery, Harbin Medical University Cancer Hospital, Harbin, China

**Keywords:** WeChat-platform based education and care program, parents of pediatric and adolescent patients with osteosarcoma, anxiety, depression, PTSD

## Abstract

**Background:**

WeChat is the main social platform in China, characterized by its versatility and ease of communication. This study aimed to explore the effect of a WeChat-platform-based education and care (WBEC) program on relieving anxiety, depression, and post-traumatic stress disorder (PTSD) in parents of pediatric and adolescent patients with osteosarcoma.

**Methods:**

In total, 48 patients and 86 parents were enrolled in this randomized, controlled study and then assigned to the WBEC program (24 patients and 45 parents) and the usual education and care (UEC) program (22 patients and 41 parents) for 6 months as a 1:1 ratio.

**Results:**

Parents in the WBEC group had lower Hospital Anxiety and Depression Scale (HADS) for anxiety (HADS-A) scores at M3 (7.8 ± 2.2 vs. 9.1 ± 2.5; *p* = 0.010) and M6 (7.7 ± 2.5 vs. 8.9 ± 2.4; *p* = 0.027) when compared to the UEC group, while anxiety rate was only decreased at M3 (43.2% vs. 63.4%; *p* = 0.049) in the WBEC group. Meanwhile, parental HADS for depression (HADS-D) scores were reduced at M3 (7.0 ± 2.0 vs. 8.0 ± 2.1; *p* = 0.047) and M6 (7.1 ± 1.9 vs. 8.0 ± 2.4; *p* = 0.045) in the WBEC group when compared with the UEC group; while depression rate remained the same. Parental Impact of Event Scale-Revised (IES-R) scores were slightly reduced among the WBEC group at M6 when compared with the UEC group [12.0 (interquartile range (IQR): 10.0–20.8] vs. 15.0 (IQR: 9.5–25.0; *p* = 0.077)], but not statistically significant.

**Conclusion:**

WeChat-platform-based education and care is considered a feasible intervention to reduce anxiety and depression in parents of pediatric and adolescent patients with osteosarcoma, while also providing mild relief from PTSD.

## Introduction

Osteosarcoma, a rare primary bone tumor and the third most common cancer in children and adolescents, is characterized by strong invasiveness and frequent metastasis (Simpson and Brown, 2018; Eaton et al., 2021). Compared with other ages, adolescents and young adults account for nearly 44% of osteosarcoma cases, and the 5-year overall survival rate is 68% in adolescent patients with osteosarcoma (Lee et al., 2021). The current optimal treatment for osteosarcoma is surgical resection (Zhu et al., 2018, 2019). However, either amputation or limb-sparing surgery combined with neoadjuvant/adjuvant chemotherapy will adversely affect the lives of childhood and adolescent patients with osteosarcoma (Solooki et al., 2018; Evans et al., 2020). Meanwhile, the use of chemotherapeutic drugs may put patients at increased risk of complications, such as vomiting, secondary malignancies, and heart failure (Zhang et al., 2018; Bhagat and Kleinerman, 2020). As a result, the parents of pediatric and adolescent patients with osteosarcoma usually bear a huge psychological burden, which may induce psychological diseases, such as anxiety, depression, and post-traumatic stress disorder (PTSD) (Yonemoto et al., 2012; Shunmugasundaram and Veeraiah, 2020). Therefore, for the parents of patients with osteosarcoma, who may also suffer from the abovementioned mental diseases, it is particularly important to find an intervention method to relieve their psychological stress.

WeChat is the mainstream social application in China with over 1.1 billion monthly active users, which is characterized by simple operations and diverse functions (Li et al., 2019; Chen et al., 2020; Su and Xiao, 2021). Due to these properties, WeChat is used as a major platform for non-drug intervention and an essential way to guide mental health not only in patients but also in the parents of pediatric and in patients with adolescent in recent years (Li et al., 2019; Liu et al., 2021; Ma et al., 2021; Yilmaz et al., 2021; Zhang et al., 2021, 2022). One study points out that WeChat-based health education helps to ease the mental state of parents of children with ventricular septal defects (Zhang et al., 2022). Meanwhile, a paper claims that the use of WeChat-based interventions effectively improves anxiety and depression in parents of infants with congenital heart disease after discharge from the hospital (Zhang et al., 2021). Moreover, a study shows that WeChat-based parenting training relieves the psychological stress in parents of autistic children during COVID-19 (Liu et al., 2021). Consequently, it could be speculated that the WeChat-based care program might also be effective for relieving the psychological problems of parents of patients with osteosarcoma. However, no study reports that.

This study designed a WeChat-platform-based education and care (WBEC) program, such as health guidance and education on discharge, information transfer and communication, information database, and online education and counseling, which aimed to explore the role of WBEC in reducing anxiety, depression, and PTSD in parents of pediatric and adolescent patients with osteosarcoma.

## Methods

### Subjects

From February 2017 to January 2021, this randomized, controlled study consecutively included a total of 46 childhood and adolescent patients with osteosarcoma and 85 parents (39 patients had two corresponding parents; while seven patients had one). The inclusion criteria were set as follows: (i) patients were diagnosed with osteosarcoma according to the National Comprehensive Cancer Network (NCCN) guideline for bone cancer (Biermann et al., 2017); (ii) patients were under the age of 20; (iii) patients underwent tumor resection; (iv) parents volunteered to participate in the study; (v) parents could use WeChat correctly; and (vi) parents were willing to be followed up regularly. The exclusion criteria were as follows: (i) patients had other hematologic malignancies or solid tumors; (ii) parents had documented mental diseases; (iii) parents were unable to fill out the scales of Hospital Anxiety and Depression Scale (HADS) and Impact of Event Scale-Revised (IES-R). The study was permitted by the Ethics Committee. All parents and children over the age of 10 signed their informed consent form.

### Random assignment

Using the blocked randomization method with a block size of four, the included subjects were randomly assigned into two groups at a ratio of 1:1 based on patients: the WBEC group had 24 patients and 45 parents and the usual education and care (UEC) group had 22 patients and 41 parents. The assignment sequence was generated using SAS 9.0 (SAS Institute, Inc., USA), then random assignment was carried out by an investigator who was blinded to the patient's information.

### Data collection

After recruitment, clinical characteristics were collected from patients, such as age, gender, tumor location, World Health Organization (WHO) classification of sarcoma, pathological fracture, Enneking stage, and surgery type. In addition, parents' clinical characteristics were also obtained, such as relationship, age, smoking status, drinking status, hypertension, hyperlipidemia, diabetes, marital status, employment status, level of education, location, and family's annual income.

### Intervention in the WBEC group

In the WBEC group, one of the investigators in the study registered a WeChat (Tencent Corporation, China) official account and a WeChat group, then the parents of patients were asked to join. The WeChat official account was a platform for patients or parents to browse information online at any time. The WeChat group was used for real-time communication and sharing information online. The intervention in the WBEC group mainly included (i) health guidance and education on discharge: health brochures of basic guidance and education were issued to parents on the day of patient's discharge, and the trained nurses were supposed to explain the contents of brochure for at least 1 h, which included post-operation care, diet health, medication administration, periodic re-examination, matters needing attention, etc.; (ii) information transfer and communication: articles, short videos or cartoons related to osteosarcoma were uploaded by investigators every week in the WeChat group to facilitate patients and parents understand the disease and help patients recover, which included post-operative rehabilitation, physical exercise, psychological health, medicine management, emergency situation management, etc., and the WeChat group was also used to supplementally explain problems and communicate with others to share post-operative rehabilitation experiences or relieve pressure; (iii) information database: the WeChat official account was served as an information sharing platform for patients or parents to browse health information related to osteosarcoma at any time (such as the consents previously sent in the WeChat group), and the information on the WeChat official account was systematic and comprehensive, which were convenient for patients and parents to consult; (iv) online education and counseling: the trained nurses were online at the WeChat official account and the WeChat group every day to help explain questions, provide psychological counseling, and help outpatient appointment, and if necessary, patients or their parents were supposed to contact the trained nurses in one-to-one chatting model (Kang and Li, 2022).

### Intervention in the UEC group

In the UEC group, patients or their parents were given a health brochure and a supplementary presentation by the trained nurses on the day of discharge, and the contents of the brochure and the presentation were the same as in the WBEC group. In addition, parents could add the researcher's phone number to communicate when needed.

### Evaluation

Hospital Anxiety and Depression Scale-anxiety (HADS-A) and HADS-depression (HADS-D) were respectively applied to evaluate the parents' anxiety and depression on the day after discharge (M0), 1 month after discharge (M1, ±1 week), 3 months after discharge (M3, ±2 weeks), and 6 months after discharge (M6, ±2 weeks). The anxiety or depression level rose as the score increased. HADS-A score >7 was considered as anxiety; while HADS-D score >7 was considered as depression (Zigmond and Snaith, 1983). Additionally, IES-R was applied to assess the parents' PTSD at M0, M3, and M6, and a higher score indicated a severer stress level (Horowitz et al., 1979; Asukai et al., 2002).

### Statistics

The sample size was estimated using PASS V.11.0 (NCSS, LLC, USA) (Wang and Sun, 2016) based on the prediction of the HADS-A score of parents at M6: WBEC group, 7.5 ± 2.0 [mean standard deviation (SD)]; UEC group, 9.0 ± 2.0 (mean SD) (Bench et al., 2015). With a power of 90% and a significant level of <0.05, the required minimum sample size was 38 in each group. Considering 10% dropouts, the final minimum sample size was increased to 41 in each group. SPSS 22.0 (IBM, USA) (Mushquash and O'Connor, 2006) and GraphPad Prism 7.02 (GraphPad Software Inc., USA) (Mitteer et al., 2018) were used for statistical analyses and figure plotting, respectively. Differences between the WBEC group and the UEC group were compared using the Mann-Whitney U test (for the skewed distributed continuous variable), t-test (for the normal distributed continuous variable), ?^2^ test, or Fisher's exact test (for the skewed distributed continuous variable). *p* < 0.05 was considered as significant.

## Results

### Study flow

**Figure 1:**
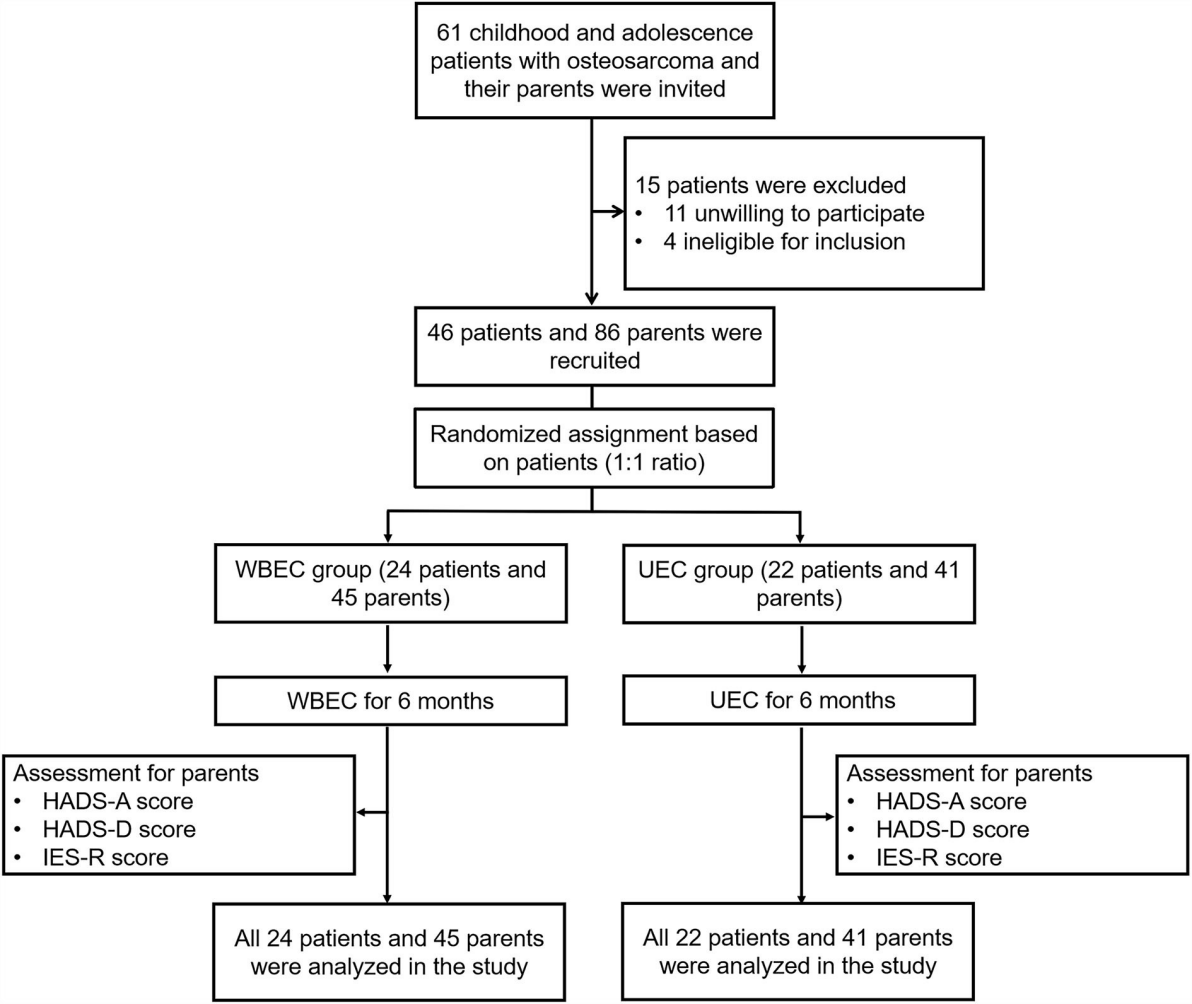
Flowchart. WBEC, WeChat-platform-based education and care program; UEC, usual education and care program; HADS-A, Hospital Anxiety and Depression Scale for anxiety; HADS-D, Hospital Anxiety and Depression Scale for depression; IES-R, Impact of Event Scale-Revised.

A total of 61 childhood and adolescent patients with osteosarcoma and their parents were invited to this study; while 15 patients were excluded, including 11 patients who were unwilling to participate and four patients who were not eligible for inclusion. The remaining 46 patients and 86 parents were randomly assigned to two groups in a 1:1 ratio based on patients. Among these, 24 patients and their 45 parents were assigned to the WBEC group and received WBEC for 6 months; whereas the other 22 patients and accordingly 41 parents were assigned to the UEC group and received UEC for 6 months. During the study, HADS-A and HADS-D scores at M0, M1, M3, and M6, as well as IES-R scores at M0, M3, and M6, were assessed to all of the parents. Finally, the data of all patients and their parents were analyzed (Figure 1).

### Clinical characteristics of patients and their parents

In terms of patients, the median age of the WBEC group and the UEC group was 13.0 [interquartile range (IQR): 9.3–14.8] years and 12.5 (IQR: 9.8–15.3) years, respectively (*p* = 0.965). There were 10 (41.7%) women and 14 (58.3%) men in the WBEC group; while 7 (31.8%) women and 15 (68.2%) men in the UEC group (*p* = 0.489). The specific clinical characteristics of enrolled patients are listed in Table 1.

**Table 1:** Clinical characteristics of patients.

**Items**	**WBEC patients (*N* = 24)**	**UEC patients (*N* = 22)**	***p*-Value**
Age (years), median (IQR)	13.0 (9.3–14.8)	12.5 (9.8–15.3)	0.965
**Gender, No. (%)**
Female	10 (41.7)	7 (31.8)	0.489
Male	14 (58.3)	15 (68.2)	
**Tumor location, No. (%)**
Femur	12 (50.0)	12 (54.5)	0.633
Tibia	9 (37.5)	9 (40.9)	
Others	3 (12.5)	1 (4.5)	
**WHO classification of sarcoma, No. (%)**
Conventional: chondroblastic	5 (20.8)	2 (9.1)	0.321
Conventional: osteoblastic	14 (58.3)	17 (77.3)	
Conventional: other	3 (12.5)	3 (13.6)	
Telangiectatic	2 (8.3)	0 (0.0)	
**Pathological fracture, No. (%)**
No	20 (83.3)	17 (77.3)	0.718
Yes	4 (16.7)	5 (22.7)	
**Enneking stage, No. (%)**
I	2 (8.3)	2 (9.1)	0.549
IIA	9 (37.5)	5 (22.7)	
IIB	13 (54.2)	15 (68.2)	
**Surgery type, No. (%)**
Limb salvage	15 (62.5)	17 (77.3)	0.277
Amputation	9 (37.5)	5 (22.7)	

WBEC, WeChat-platform based education and care program; UEC, usual education and care program; IQR, interquartile range; WHO, World Health Organization.

Regarding parents, there were 23 (52.3%) mothers and 21 (47.7%) fathers in the WBEC group, as well as 22 (53.7%) mothers and 19 (46.3%) fathers in the UEC group (*p* = 0.898). The median age of the WBEC group was 41.0 (IQR: 37.0–46.0) years; while it was 41.0 (IQR: 37.0–43.5) for the UEC group (*p* = 0.231). Moreover, the detailed clinical characteristics of parents are listed in Table 2.

**Table 2:** Clinical characteristics of parents.

**Items**	**WBEC parents (*N* = 44)**	**UEC parents (*N* = 41)**	***p*-Value**
**Relation, No. (%)**			0.898
Mother	23 (52.3)	22 (53.7)	
Father	21 (47.7)	19 (46.3)	
Age (years), median (IQR)	41.0 (37.0–46.0)	41.0 (37.0–43.5)	0.231
**Smoke status, No. (%)**			0.800
Never smoke	17 (38.6)	17 (41.5)	
Former smoker	9 (20.5)	10 (24.4)	
Current smoker	18 (40.9)	14 (34.1)	
**Drink status, No. (%)**			0.697
No	25 (56.8)	25 (61.0)	
Yes	19 (43.2)	16 (39.0)	
**Hypertension, No. (%)**			0.265
No	36 (81.8)	37 (90.2)	
Yes	8 (18.2)	4 (9.8)	
**Hyperlipidemia, No. (%)**			1.000
No	41 (93.2)	39 (95.1)	
Yes	3 (6.8)	2 (4.9)	
**Diabetes, No. (%)**			0.669
No	42 (95.5)	38 (92.7)	
Yes	2 (4.5)	3 (7.3)	
**Marriage status, No. (%)**			1.000
Married	40 (90.9)	38 (92.7)	
Divorced/widowed	4 (9.1)	3 (7.3)	
**Employment status, No. (%)**			0.885
Employed	37 (84.1)	34 (82.9)	
Unemployed	7 (15.9)	7 (17.1)	
**Level of education, No. (%)**			0.850
Primary school or less	2 (4.5)	3 (7.3)	
High school	17 (38.6)	18 (43.9)	
Undergraduate	21 (47.7)	16 (39.0)	
Graduate or above	4 (9.1)	4 (9.8)	
**Location, No. (%)**			0.511
Urban	40 (90.9)	35 (85.4)	
Rural	4 (9.1)	6 (14.6)	
**Family annual income, No. (%)**			0.726
10,000–29,999 RMB	10 (22.7)	12 (29.3)	
30,000–49,999 RMB	17 (38.6)	16 (39.0)	
≥50,000 RMB	17 (38.6)	13 (31.7)	

WBEC, WeChat-platform based education and care program; UEC, usual education and care program; IQR, interquartile range.

### Comparison of parental anxiety between the WBEC and UEC groups

**Figure 2:**
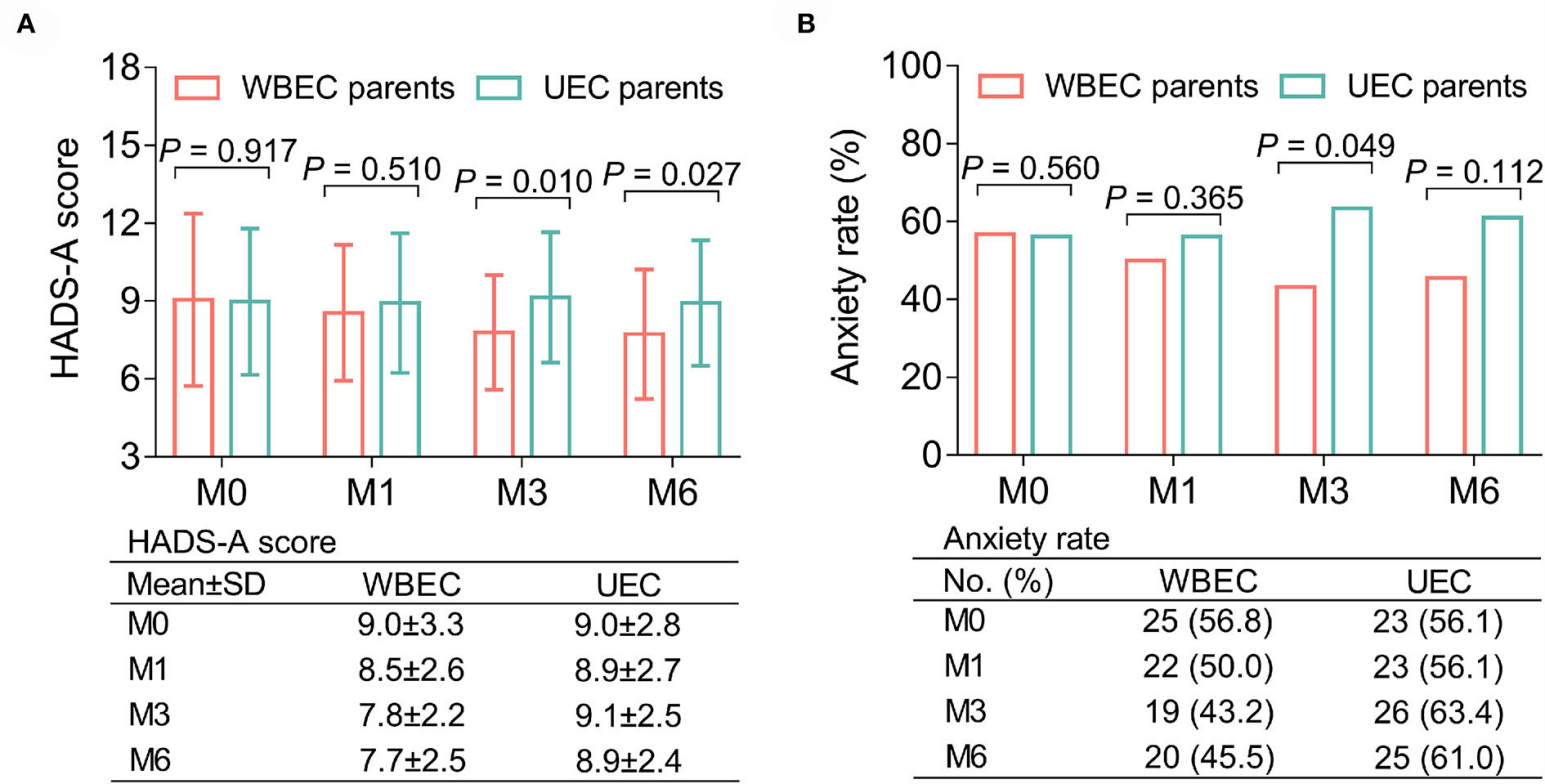
Effect of WBEC on anxiety in parents of pediatric and adolescent patients with osteosarcoma. Comparison of HADS-A score **(A)** and anxiety rate **(B)** at M0, M1, M3, and M6 between parents in WBEC group and UEC group. WBEC, WeChat-platform-based education and care program; UEC, usual education and care program; HADS-A, Hospital Anxiety and Depression Scale for anxiety; M, month.

Parents in the WBEC group exhibited lower HADS-A scores at M3 (7.8 ± 2.2 vs. 9.1 ± 2.5; *p* = 0.010) and M6 (7.7 ± 2.5 vs. 8.9 ± 2.4; *p* = 0.027) when compared with UEC group; while no differences of HADS-A scores were observed between parents in the WBEC and UEC groups at M0 (9.0 ± 3.3 vs. 9.0 ± 2.8) and M1 (8.5 ± 2.6 vs. 8.9 ± 2.7; both *p* > 0.05; Figure 2A). Regarding anxiety rate, it was decreased in parents of WBEC group when compared with UEC group at M3 (43.2 vs. 63.4%; *p* = 0.049) but no differences were found between the two groups of parents at M0 (56.8 vs. 56.1%), M1 (50.0 vs. 56.1%), or M6 (45.5 vs. 61.0%; all *p* > 0.05; Figure 2B).

### Comparison of parental depression between the WBEC and UEC groups

**Figure 3:**
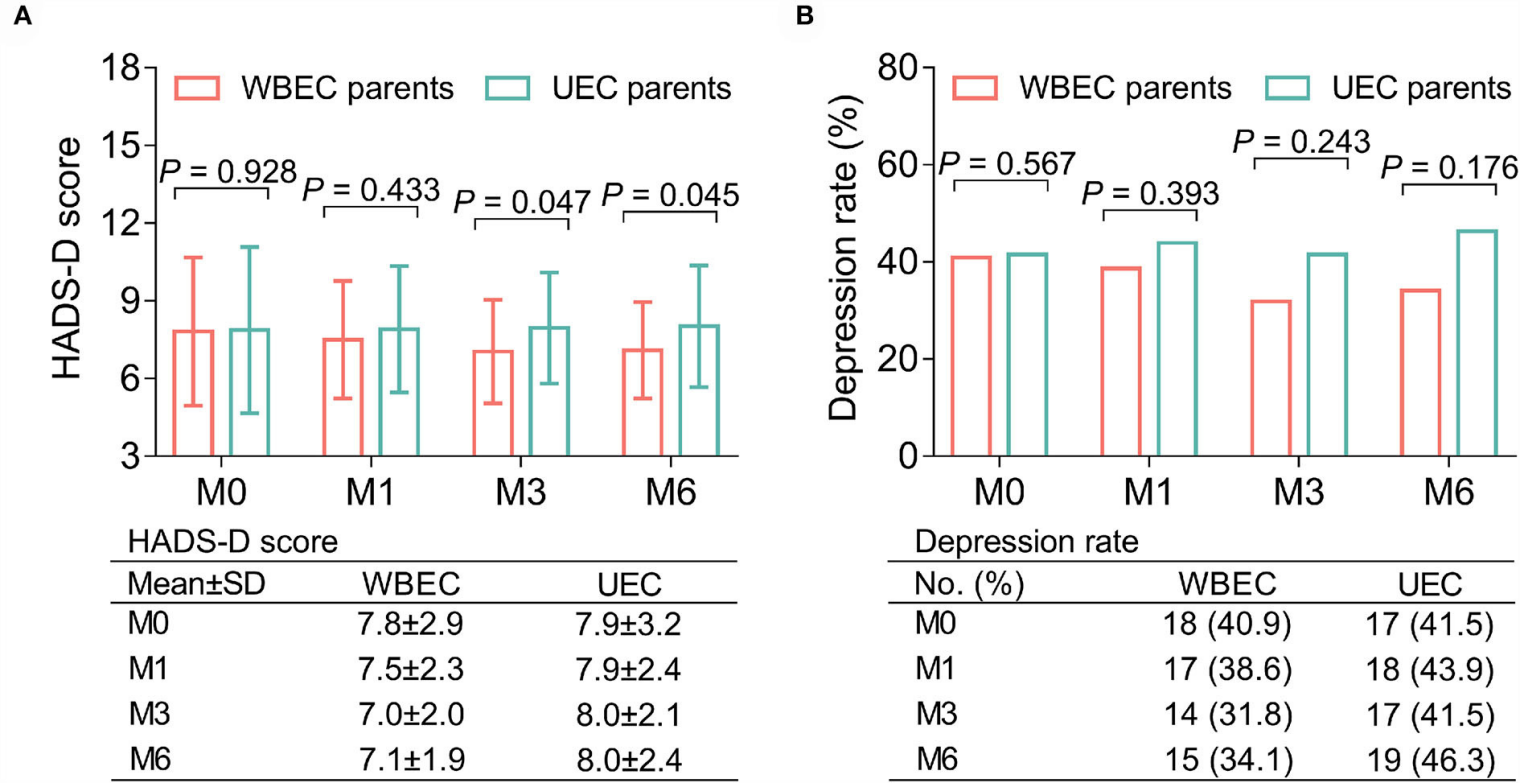
Effect of WBEC on depression in parents of pediatric and adolescent patients with osteosarcoma. Comparison of HADS-D score **(A)** and depression rate **(B)** between parents in WBEC group and UEC group at M0, M1, M3, and M6. WBEC, WeChat-platform-based education and care program; UEC, usual education and care program; HADS-D, Hospital Anxiety and Depression Scale for depression; M, month.

Parental HADS-D scores were lower in the WBEC group than those in the UEC group at M3 (7.0 ± 2.0 vs. 8.0 ± 2.1; *p* = 0.047) and M6 (7.1 ± 1.9 vs. 8.0 ± 2.4; *p* = 0.045); while no differences were observed at M0 (7.8 ± 2.9 vs. 7.9 ± 3.2) and M1 (7.5 ± 2.3 vs. 7.9 ± 2.4; both *p* > 0.05; Figure 3A). Meanwhile, there were no differences in depression rates between the two groups of parents at all time points (all *p* > 0.05; Figure 3B).

### Comparison of parental IES-R scores between WBEC and UEC groups

**Figure 4:**
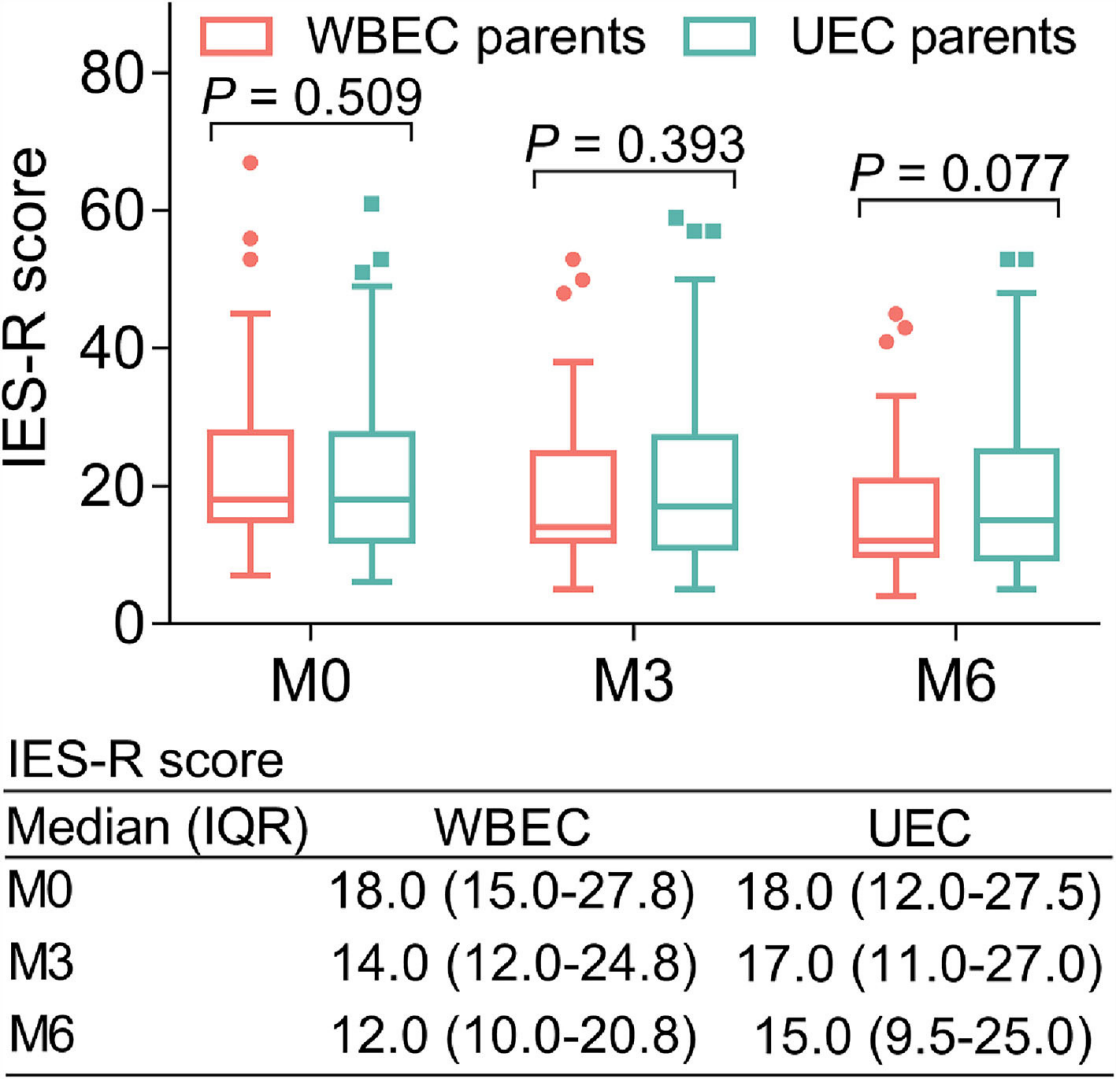
Effect of WBEC on PTSD in parents of pediatric and adolescent patients with osteosarcoma. Comparison of IES-R score at M0, M3, and M6 between parents in WBEC group and UEC group. WBEC, WeChat-platform-based education and care program; UEC, usual education and care program; IES-R, Impact of Event Scale-Revised.

No difference of IES-R score was found at M0 [18.0 (IQR: 15.0–27.8) vs. 18.0 (IQR: 12.0–27.5); *p* > 0.05] and M3 [14.0 (IQR: 12.0–24.8) vs. 17.0 (IQR: 11.0–27.0); *p* = 0.393] between parents in the WBEC and UEC groups. However, the IES-R score showed a slight decrease in parents of the WBEC group when compared with parents in the UEC group at M6 [12.0 (IQR: 10.0–20.8) vs. 15.0 (IQR: 9.5–25.0); *p* = 0.077] but did not reach statistical significance (Figure 4).

### Subgroup analyses of HADS score, anxiety, depression, and IES-R score

In the mother (*p* = 0.027), married (*p* = 0.022), employed (*p* = 0.034), and family annual income ≥50,000 RMB (*p* = 0.014) parents, HADS-A scores at M6 were reduced in WBEC group when compared with UEC group. Meanwhile, in the mother (*p* = 0.033), undergraduate or above (*p* = 0.036), rural area (*p* = 0.031), and annual family income of 30,000–49,999 RMB (*p* = 0.038) parents, HADS-D scores at M6 in WBEC group were lower than those in the UEC group. In the end, in the father (*p* = 0.011) and divided/widowed status (*p* = 0.031) of parents in the WBEC group, IES-R scores at M6 were decreased, compared with the UEC group. Detailed subgroup analysis could be checked in Table 3.

**Table 3:** Subgroup analysis of HADS score, anxiety, depression and IES-R score.

**Items**	**HADS-A score (M6)**		**Anxiety rate (M6)**		**HADS-D score (M6)**		**Depression rate (M6)**		**IES-R score (M6)**	
	**Mean ±SD**	***p*-Value**	**No. (%)**	***p*-Value**	**Mean ±SD**	***p*-Value**	**No. (%)**	***p*-Value**	**Median (IQR)**	***p*-Value**
**Relation**
Mother		**0.027**		0.088		**0.033**		0.299		0.883
WBEC parents	7.9 ± 2.4		11 (47.8)		7.0 ± 1.8		7 (30.4)		14.0 (11.0–25.0)	
UEC parents	9.6 ± 2.5		16 (72.7)		8.4 ± 2.6		10 (45.5)		16.0 (8.8–26.0)	
Father		0.410		0.775		0.594		0.554		**0.011**
WBEC parents	7.5 ± 2.6		9 (42.9)		7.2 ± 1.9		8 (38.1)		11.0 (8.0–14.0)	
UEC parents	8.2 ± 2.2		9 (47.4)		7.6 ± 2.1		9 (47.4)		15.0 (10.0–23.0)	
**Marry status**
Married		**0.022**		0.170		0.066		0.267		0.231
WBEC parents	7.7 ± 2.3		18 (45.0)		7.0 ± 1.8		13 (32.5)		12.5 (10.0–20.8)	
UEC parents	8.9 ± 2.5		23 (60.5)		7.9 ± 2.3		17 (44.7)		15.0 (9.0–23.0)	
Divorced/widowed		0.882		1.000		0.401		1.000		**0.031**
WBEC parents	8.3 ± 4.3		2 (50.0)		7.8 ± 2.5		2 (50.0)		11.0 (7.3–18.5)	
UEC parents	8.7 ± 1.5		2 (66.7)		9.7 ± 3.1		2 (66.7)		26.0 (26.0–NA)	
**Employment status**
Employed		**0.034**		0.190		0.054		0.209		0.172
WBEC parents	7.5 ± 2.4		16 (43.2)		6.9 ± 1.9		11 (29.7)		12.0 (10.0–20.5)	
UEC parents	8.8 ± 2.4		20 (58.8)		7.9 ± 2.3		15 (44.1)		16.0 (9.0–23.3)	
Unemployed		0.537		1.000		0.588		1.000		0.223
WBEC parents	8.9 ± 2.6		4 (57.1)		8.1 ± 1.2		4 (57.1)		12.0 (9.0–22.0)	
UEC parents	9.7 ± 2.4		5 (71.4)		8.7 ± 2.4		4 (57.1)		15.0 (12.0–36.0)	
**Level of education**
High school or less		0.099		0.218		0.443		0.427		0.296
WBEC parents	7.7 ± 2.6		9 (47.4)		6.8 ± 2.0		5 (26.3)		12.0 (8.0–21.0)	
UEC parents	9.1 ± 2.7		14 (66.7)		7.2 ± 1.7		8 (38.1)		15.0 (9.0–26.5)	
Undergraduate or above		0.166		0.463		**0.036**		0.316		0.092
WBEC parents	7.8 ± 2.5		11 (44.0)		7.3 ± 1.8		10 (40.0)		12.0 (10.5–20.5)	
UEC parents	8.8 ± 2.2		11 (55.0)		8.9 ± 2.7		11 (55.0)		17.0 (12.5–22.8)	
**Location**
Urban		0.152		0.422		0.099		0.471		0.076
WBEC parents	7.7 ± 2.5		18 (45.0)		7.3 ± 1.8		15 (37.5)		12.0 (10.0–20.8)	
UEC parents	8.5 ± 2.1		19 (54.3)		8.1 ± 2.5		16 (45.7)		17.0 (10.0–24.0)	
Rural		0.069		0.133		**0.031**		0.200		0.668
WBEC parents	7.8 ± 2.5		2 (50.0)		5.0 ± 0.8		0 (0.0)		12.0 (8.5–23.8)	
UEC parents	11.3 ± 2.7		6 (100.0)		7.3 ± 1.6		3 (50.0)		13.0 (8.8–38.8)	
**Family annual income**
10,000–29,999 RMB		0.526		1.000		0.428		0.691		0.644
WBEC parents	9.0 ± 2.2		7 (70.0)		7.3 ± 2.4		4 (40.0)		12.5 (9.5–28.3)	
UEC parents	9.7 ± 2.6		9 (75.0)		8.3 ± 3.0		6 (50.0)		16.0 (9.8–22.8)	
30,000–49,999 RMB		0.467		0.849		**0.038**		0.387		0.678
WBEC parents	8.2 ± 2.9		9 (52.9)		7.1 ± 1.8		7 (41.2)		16.0 (11.0–23.0)	
UEC parents	8.9 ± 2.5		9 (56.3)		8.6 ± 2.2		9 (56.3)		16.5 (10.5–32.0)	
≥50,000 RMB		**0.014**		0.132		0.827		0.698		0.068
WBEC parents	6.5 ± 1.5		4 (23.5)		6.9 ± 1.7		4 (23.5)		11.0 (7.5–14.0)	
UEC parents	8.2 ± 2.1		7 (53.8)		7.1 ± 1.6		4 (30.8)		15.0 (8.5–23.0)	

HADS, Hospital Anxiety and Depression Scale; IES-R, Impact of Event Scale-Revised; HADS-A, Hospital Anxiety and Depression Scale-Anxiety; M6, at six months; HADS-D, Hospital Anxiety and Depression Scale-Depression; IQR, interquartile range. The bold values mean statistical significance (P < 0.05).

## Discussion

WeChat-based interventions are currently receiving intensive attention as an innovative approach to deliver rehabilitation programs (Dorje et al., 2018). Some studies report that WeChat-based interventions are capable of reducing anxiety and depression in child and adolescent patients' parents (Liu et al., 2021; Zhang et al., 2021, 2022). Similarly, the WBEC program was set up in this study, such as health guidance and education on discharge, information transfer and communication, information database, and online education and counseling. It was observed that the level of anxiety and depression in parents of WBEC was lower than that in UEC parents. The possible explanations might be that (i) WeChat is characterized by convenience, concentration, and flexibility, which allows parents of the patients with osteosarcoma to have access to disease-related knowledge after the patients are discharged from the hospital, thus helping their children with rehabilitation, physical exercise, and medication management, etc. (Luo et al., 2019). (ii) The establishment of the information database provided parents with a platform to actively learn about osteosarcoma-related information, which indicated that they might be more prepared to manage their children (Wang et al., 2020). (iii) Through the one-to-one or group chat mode of WeChat, parents could get in touch with the nursing staff timely to obtain professional advice; they could also communicate with other members to exchange experiences, encourage each other, and reduce negative emotions (Luo et al., 2019).

The current primary treatments for PTSD consist of psychological intervention and drug therapy. Psychological interventions, such as cognitive processing therapy (CPT), cognitive behavioral therapy (CBT), and cognitive restructuring (CR), are considered effective tools. However, the recommendations of current guidelines appear to be empirically inferred, so the psychological interventions mentioned above have certain limitations (Miao et al., 2018). In terms of medical treatment, a review suggests that medicinal cannabis may be beneficial; whereas this method also has many hidden dangers, such as that it may lead to addiction, it is not suitable for adolescents, and it may also cause anxiety, paranoia, and cognitive impairment (Sarris et al., 2020). Based on the above considerations, a WBEC program was initiated in this study to alleviate PTSD. It was clear that WBEC could slightly reduce PTSD in parents of pediatric and adolescent patients with osteosarcoma, but this did not reach a statistical significance. The reasons could be that (i) WBEC removed the barriers of time, finance, and space, which allowed parents of patients with osteosarcoma to receive education and care in familiar surroundings (Ma et al., 2018; Luo et al., 2019). (ii) Efficient information transmission and communication allowed parents to communicate with nursing staff promptly to obtain professional guidance and advice. (iii) The reason for the lack of statistical significance may be due to the short period of this study.

Simultaneously, it was discovered that for some factors, such as motherhood, being married, being employed, and having a family annual income ≥50,000 RMB, WBEC was more effective than UEC in alleviating anxiety. At the same time, in factors, such as motherhood, living in a rural area, holding an undergraduate or above degree, and having a family annual income of 30,000–49,999 RMB, WBEC was more efficient than UEC in alleviating depression. Moreover, in factors, such as fatherhood and being divorced/widowed, WBEC was more effective than UEC in reducing PTSD. As a result, these findings could provide some referential importance for the stratified management in parents of pediatric and adolescent patients with osteosarcoma. However, further studies are needed to confirm these findings.

It should be noted that this study had certain limitations. Firstly, this was a single-center study, which would result in selection bias. Consequently, a multicenter study conducted in diverse regions could be considered in further studies. Secondly, the follow-up time was short, so the long-term impact of the WBEC program on parents of pediatric and adolescent patients with osteosarcoma was still mysterious. Thirdly, this study concentrated on the effect of WBEC on parents of pediatric and adolescent patients with osteosarcoma, but it remains to be explored whether WBEC would be effective for parents of other childhood and adolescent patients with cancer. Fourthly, in this study, the minimum sample size was calculated and patients were further included according to this sample size, however, further studies could consider expanding the sample size to further validate the findings. Fifthly, the HADS score for evaluating anxiety and depression was a self-assessed questionnaire, which might exist an assessment bias. Sixthly, this study mainly investigated the WBEC program for parents of pediatric and adolescent patients with osteosarcoma; however, anxiety and depression would occur in patients themselves as well; therefore, further study could explore the value of WBEC for pediatric and adolescent patients with osteosarcoma. Seventhly, some compounding factors might exist in this study, such as technological differences, single parents, and educational levels, which might lead to biased results.

In conclusion, WBEC not only relieves anxiety and depression but also alleviates PTSD to a certain extent in parents of pediatric and adolescent patients with osteosarcoma.

## Data availability statement

The original contributions presented in the study are included in the article/supplementary material, further inquiries can be directed to the corresponding author.

## Ethics statement

The studies involving human participants were reviewed and approved by Harbin Medical University Cancer Hospital. The patients/participants provided their written informed consent to participate in this study.

## Author contributions

HL conceived and designed the study. JW and JM collected, analyzed the data, and wrote the manuscript. JW and HL prepared the figures, tables, and revised the manuscript. All authors read and approved the submitted version.

## Conflict of interest

The authors declare that the research was conducted in the absence of any commercial or financial relationships that could be construed as a potential conflict of interest.

## Publisher's note

All claims expressed in this article are solely those of the authors and do not necessarily represent those of their affiliated organizations, or those of the publisher, the editors and the reviewers. Any product that may be evaluated in this article, or claim that may be made by its manufacturer, is not guaranteed or endorsed by the publisher.
